# Role of intracellular and extracellular annexin A1 in migration and invasion of human pancreatic carcinoma cells

**DOI:** 10.1186/1471-2407-14-961

**Published:** 2014-12-16

**Authors:** Raffaella Belvedere, Valentina Bizzarro, Ada Popolo, Fabrizio Dal Piaz, Michele Vasaturo, Paola Picardi, Luca Parente, Antonello Petrella

**Affiliations:** Department of Pharmacy, University of Salerno, via Giovanni Paolo II 132, 84084 Fisciano, SA Italy

**Keywords:** Annexin A1, Pancreatic cancer, Formyl peptide receptors, Cell migration, Cell invasion

## Abstract

**Background:**

Annexin A1 (ANXA1), a 37 kDa multifunctional protein, is over-expressed in tissues from patients of pancreatic carcinoma (PC) where the protein seems to be associated with malignant transformation and poor prognosis.

**Methods:**

The expression and localization of ANXA1 in MIA PaCa-2, PANC-1, BxPC-3 and CAPAN-2 cells were detected by Western Blotting and Immunofluorescence assay. Expression and activation of Formyl Peptide Receptors (FPRs) were shown through flow cytometry/PCR and FURA assay, respectively. To investigate the role of ANXA1 in PC cell migration and invasion, we performed *in vitro* wound-healing and matrigel invasion assays.

**Results:**

In all the analyzed PC cell lines, a huge expression and a variable localization of ANXA1 in sub-cellular compartments were observed. We confirmed the less aggressive phenotype of BxPC-3 and CAPAN-2 compared with PANC-1 and MIA PaCa-2 cells, through the evaluation of Epithelial-Mesenchymal Transition (EMT) markers. Then, we tested MIA PaCa-2 and PANC-1 cell migration and invasiveness rate which was inhibited by specific ANXA1 siRNAs. Both the cell lines expressed FPR-1 and -2. Ac2-26, an ANXA1 mimetic peptide, induced intracellular calcium release, consistent with FPR activation, and significantly increased cell migration/invasion rate. Interestingly, in MIA PaCa-2 cells we found a cleaved form of ANXA1 (33 kDa) that localizes at cellular membranes and is secreted outside the cells, as confirmed by MS analysis. The importance of the secreted form of ANXA1 in cellular motility was confirmed by the administration of ANXA1 blocking antibody that inhibited migration and invasion rate in MIA PaCa-2 but not in PANC-1 cells that lack the 33 kDa ANXA1 form and show a lower degree of invasiveness. Finally, the treatment of PANC-1 cells with MIA PaCa-2 supernatants significantly increased the migration rate of these cells.

**Conclusion:**

This study provides new insights on the role of ANXA1 protein in PC progression. Our findings suggest that ANXA1 protein could regulate metastasis by favouring cell migration/invasion intracellularly, as cytoskeleton remodelling factor, and extracellularly like FPR ligand.

## Background

Pancreatic carcinoma (PC) is one of the most aggressive gastrointestinal malignancies worldwide, with poor prognosis [1] and a 5-year survival rate of 3-5% [2].

The current standard treatment for patients affected by PC is surgery, radiations and drugs as gemcitabine and TS-1 [3]. However, most patients present a marked resistance to chemo- and radiotherapy that impacts their therapeutic effects. Moreover, PC invades progressively and metastasizes to liver and lymph nodes during early stages without remarkable symptoms so that, surgery is not an option for the majority of these patients that present rapid relapse [4, 5].

Tumour metastases are the most common causes of death in cancer patients and represent the utmost challenge for cancer treatment. In particular cell migration and invasion play a crucial role in the progression of cancer since their deregulation causes tumour metastasis [6]. Thus, a better understanding of the mechanisms underlying these processes is important for the development of novel anticancer agents in order to improve clinical outcome.

Annexin A1 (ANXA1) is a key member of the A subfamily and belongs to the multi-gene family of annexins. ANXA1 exhibits calcium-mediated phospholipid binding properties and participates in many physiopathological processes, including inhibition of cell proliferation, anti-inflammatory effects, regulation of cell migration, differentiation and death [7].

ANXA1 appearance is in a tissue- and tumour-specific manner and its anomalous expression is closely related to cancer progression [8, 9]. Up-regulated ANXA1 expression was correlated with tumour progression in urothelial carcinoma, glioma, colon carcinoma and lung squamous carcinoma, whereas down-regulated ANXA1 expression was observed in prostate, oral and gastric cancer progression. This suggests that ANXA1 influences cancer progression in different ways and that it may have different sub-cellular localizations that determine its functions [10].

One of this ways could be due to ANXA1 well known ability to bind F-actin in a Ca^2+^-dependent manner since the protein has been found to accumulate concomitantly with the appearance of F-actin at the ruffles and at the cell-cell contacts in several biological systems [11]. However, the relevance and the significance of this property remain unclear to date, also in cancer cells where the motility is driven by reorganization of the cytoskeleton and of the contacts between the cell and the matrix.

ANXA1 has been shown to localize to the cell surface of various cell types where it is thought to be important in biological functions as myoblast and skin fibroblast migration and cytoskeleton reorganization [12, 13]. The extracellular form of ANXA1 has been as well described to play a role in cancer cell invasion and metastasis. The most significant example is breast cancer, where the pro-invasive effect of ANXA1 is triggered by the interaction with the Formyl Peptide Receptors (FPRs) [14]. The regulatory action on cell surface by extracellular ANXA1 is indeed thought to be mediated by signalling through FPRs [12, 13, 15]. FPRs are G-protein coupled chemoattractant receptors, which can sense gradients of bacterial peptides such as formyl-Methionine-Leucine-Phenylalanine (fMLP) and thereby direct leukocytes towards sites of bacterial infection [16]. Three human FPR family members have been identified including FPR-1, FPR-2, and FPR-3. Ligand binding to FPR activates a number of downstream effector enzymes including phospholipase C, catalyzing the cleavage of phosphatidyl inositol 4,5-biphosphate into secondary messengers inositol 1,4,5-triphosphate and diacylglycerol leading to calcium mobilization and activation of protein kinase C (PKC) [16–18].

Previous studies have shown that tumour cells from highly malignant human glioma specimens express FPR-1 [19]; subsequently it has been shown that ANXA1 released by necrotic human glioblastoma cells was able to stimulate tumour cell growth through FPR-1 [20]. Interestingly, it has also been shown that the translocation of ANXA1 to the cell surface is partially responsible for promoting cell migration and invasion in colorectal carcinoma cell line SKCO-15, indicating an autocrine/paracrine role for membrane ANXA1 [21].

Despite the identification of ANXA1 as one of the several proteins that is differentially expressed during the progression of tumours to more malignant states, a functional role for ANXA1 in PC advancing is lacking. The aim of our study is to investigate the role of ANXA1 in human PC progression, in particular we focused on its involvement in cancer cell migration and invasiveness.

## Methods

### Cell cultures

MIA PaCa-2, human PC cells, were cultured in DMEM (Lonza) containing L-Glutamine 2 mM, 10% heat-inactivated fetal bovine serum (FBS; Lonza) and 2,5% heat inactivated horse serum (HS; Lonza). PANC-1, human pancreatic epithelioid carcinoma cells, were kept in DMEM containing L-Glutamine 2 mM and 10% heat-inactivated fetal bovine serum (FBS; Lonza). BxPC-3, human pancreatic adenocarcinoma cells, were cultured in RPMI 1640 (Lonza) containing 10% heat-inactivated fetal bovine serum (FBS; Lonza). CAPAN-2, human PDAC cells, were kept in McCoy’s 5a Medium Modified (Lonza) with 10% heat-inactivated fetal bovine serum (FBS; Lonza). All the media were supplemented with antibiotics (10000 U/ml penicillin and 10 mg/ml streptomycin; Lonza). Cell lines were purchased from ATCC (Rockville, USA) and were grown at 37°C in 5% CO_2_ -95% air humidified atmosphere.

### Cytosol and membrane extracts

MIA PaCa-2 and PANC-1 cells were washed twice with PBS, detached with trypsin-EDTA 1× in PBS (Euroclone), harvested in PBS and centrifuged for 5 minutes at 600 × g at 4°C. After that, the pellets were resuspended in 4 ml of lysis buffer (Tris HCl 20 mM, pH 7,4; sucrose 250 mM; DTT 1 mM; protease inhibitors, EDTA 1 mM in water), sonicated (5 seconds pulse - 9 seconds pause for 2 minutes, amplitude 42%) and then centrifuged at 4°C for 10 minutes, at 5000 × g. The obtained supernatants were ultra-centrifuged for 1 hour at 100000 × g at 4°C, until to get new supernatants that represent cytosol extracts. Each resulting pellet was resuspended in 4 ml of lysis buffer and ultra-centrifuged for 1 hour at 100000 × g at 4°C. The pellets were then resuspended in 250 μl of solubilization buffer (Tris HCl 20 mM, pH 7,4; DTT 1 mM; EDTA 1 mM; Triton X-100 1%, in water) and left overnight on orbital shaker at 4°C. After that, the solution was centrifuged for 30 minutes at 50000 × g at 4°C: the supernatants represent membrane extracts. To detect membrane expression of ANXA1 we also use an EDTA Wash method, as previously described [22].

### Nuclear extracts

MIA PaCa-2 and PANC-1 cells were washed twice with PBS, detached with trypsin-EDTA 1× in PBS (Euroclone), harvested in PBS and centrifuged for 5 minutes at 600 × g at 4°C. The pellets were resuspended in 500 μl of buffer A (Hepes pH 7.9 10 mM, EDTA pH 8.0 1 mM, KCl 60 mM, N-P40 0.2%, DTT 1 mM, PMSF 1 mM, protease inhibitors) and then left on ice for 10 minutes. After that, the samples were centrifuged at 660 × g for 5 minutes at 4°C, resuspended in 50 μl of buffer B (Tris HCl pH 7.8 250 mM, KCl 60 mM, DTT 1 mM, PMSF 2 mM, glycerol 20% v/v in PBS) and centrifuged again at 9500 × g for 15 minutes at 4°C. The obtained pellets were resuspended in 100 μl of buffer C (Hepes pH 7.9 10 mM, EDTA pH 8.0 1 mM, KCl 60 mM, DTT 1 mM, PMSF 1 mM, protease inhibitors) and centrifuged at 660 × g for 5 minutes at 4°C. The samples were then washed twice with 1 ml of buffer C, resuspended in 50 μl of buffer B and exposed to 3 cycles of freeze/thawing. Finally, the samples were centrifuged at 9500 × g for 15 minutes at 4°C: the pellets represent the nuclear extracts.

### Supernatant analysis

Cell growth media were harvested, frozen at -80°C and lyophilized. Dried samples were suspended in lysis buffer containing protease inhibitors and left at 4°C for 30 minutes. After centrifugation, the supernatants were filtered through Amicon Ultra-15, PLTK Ultracel-PL Membrane, 10 kDa (Millipore). The filtrates were loaded on a Chromabond HR-X micro-column (Macherey-Nagel) and eluted with 70% ACN and 95% ACN. Eluted samples were analyzed by LC/MS/MS using an Orbitrap XL instrument (Thermo Scientific) as reported elsewhere [23].

### Western blotting analysis

Expression of ANXA1 was examined by SDS-PAGE. Total intracellular proteins were extracted from the cells by freeze/thawing in lysis buffer containing protease inhibitors. Protein content was estimated according to Biorad protein assay (BIO-RAD). Samples (20 μg protein) were loaded onto 10% denaturing-polyacrylamide gel and separated by SDS-PAGE. The separated proteins were then transferred electrophoretically to nitrocellulose membranes (Immobilon-NC, Millipore). Membranes were blocked with 5% non-fat dry milk in TBS-Tween 20 (0.1% v/v) and then incubated overnight at 4°C with the primary antibodies. Proteins were visualized using the enhanced chemioluminescence detection system (Amersham Pharmacia Biotech) after incubation overnight at 4°C with primary polyclonal antibody against ANXA1 (1:10000; Invitrogen) and monoclonal a-tubulin (1:1000; Sigma-Aldrich) and then at RT with an appropriate secondary rabbit or mouse antibody (1:5000; Sigma-Aldrich). Immunoreactive protein bands were detected by chemioluminescence using enhanced chemioluminescence reagents (ECL; Amersham), the blots were exposed and analyzed to Las4000 (GE Healthcare Life Sciences).

### siRNA transfection

The 4, 6, 7 and 8 siRNA sequences against ANXA1 were purchased from Qiagen and used at a final concentration of 5 nM. siRNA Oligo-Scrambled (Santa Cruz Biotechnology) was used as control at the same concentration. siRNAs were transfected using Lipofectamine 2000 Reagent (Life technologies Corporation), according to the manufacturer′s instructions. Cells were harvested after 72 hours from transfection.

### Confocal microscopy

After the specific time of incubation, MIA PaCa-2, PANC-1, BxPC-3 and CAPAN-2 cells were fixed in p-formaldehyde (4% v/v in PBS) for 5 minutes. The cells were permeabilized in Triton X-100 (0.5% v/v in PBS) for 5 minutes, and then incubated in goat serum (20% v/v PBS) for 30 minutes, and with rabbit anti-ANXA1 antibody (1:100; Invitrogen), mouse anti-FAK (1:100; BD Transduction Laboratories), mouse anti-E-cadherin (1:250; Santa Cruz Biotechnology) and/or mouse anti-vimentin (1:500; Santa Cruz Biotechnology) overnight at 4°C. After two washing steps with PBS, cells were incubated with anti-rabbit and/or anti-mouse AlexaFluor (488 and/or 555; 1:1000; Molecular Probes) for 2 hours at RT and then with FITC-conjugated anti-F-actin (5 μg/ml; Phalloidin-FITC, Sigma) for 30 minutes at RT in the dark. The coverslips were mounted in glycerol (40% v/v PBS). A Zeiss LSM 710 Laser Scanning Microscope (Carl Zeiss MicroImaging GmbH) was used for data acquisition. To detect nucleus, samples were excited with a 458 nm Ar laser. A 555 nm He-Ne laser was used to detect emission signals from ANXA1 stain. Samples were vertically scanned from the bottom of the coverslip with a total depth of 5 mm and a 63× (1.40 NA) Plan-Apochromat oil-immersion objective. Images were generated with Zeiss ZEN Confocal Software (Carl Zeiss MicroImaging GmbH).

### Flow cytometry

MIA PaCa-2 and PANC-1 cells were harvested at a number of 1 × 10^6^ and centrifuged at 30000 × g for 5 minutes. The pellets were then incubated on ice for 1 hour in 100 μl of PBS containing a primary polyclonal antibody against FPR-1 (1:500, Santa Cruz Biotechnology) or a primary monoclonal antibody against FPR-2 (1:100, Genovac). After that, MIA PaCa-2 and PANC-1 cells were washed twice and incubated on ice for 1 hour in 100 μl of PBS containing AlexaFluor 488 anti-rabbit (1:1000; Molecular Probes) or AlexaFluor 488 anti-mouse (1:1000; Molecular Probes). The cells were analyzed with Becton Dickinson FACScan flow cytometer using the Cells Quest program.

### PCR

MIA PaCa-2 and PANC-1 cells were seeded at an initial density of 1 × 10^6^ in a 100 mm Petri dish and incubated for 48 hours in growth medium allowing cells to reach 90% confluency. Total RNA was extracted from cells using Trizol (Invitrogen) [24]. Total RNA (5 μg) was used to synthesize cDNA using a reverse transcription kit (Roche). PCR was conducted by using the following primers:

FPR-1 primer pair: (fwd 5’-CAA GAT GGA GAC AAA TTC CTC TC-3’) and (rev 3’-GAG CAG AGC CAT CAC CCA GGG CCC AA-5’);

FPR-2 primer pair: (fwd 5’-CTG TAC TTT CAA CTT TGC ATC C-3’) and (rev 3’-ATT TCC CAA CTC CAC TTA CC-5’);

The predicted FPR-1 and FPR-2 products are 469 bp and 773 bp, respectively. The FPR-1 and FPR-2 genes were amplified using PCR under the following conditions: pre-denaturation at 94°C for 2 minutes, 35 cycles of denaturation at 94°C for 30 seconds, annealing at 60°C for 30 seconds, extension at 72°C for 30 seconds and a final extension at 72°C for 10 minutes. The products were stored at 4°C. A portion (5 μl) of the PCR product was electrophoresed on a 1% agarose gel in a Tris-acetate-EDTA buffer. The gel was stained with ethidium bromide and was scanned and analysed to Las4000 (GE Healthcare Life Sciences).

### Measurement of intracellular Ca^2+^ signalling

Intracellular Ca^2+^ concentrations [Ca^2+^] were measured using the fluorescent indicator dye Fura 2-AM, the membrane-permeant acetoxymethyl ester form of Fura 2, as previously described [25], with minor revisions. Briefly, MIA PaCa-2 and PANC-1 cells (5 × 10^3^/multiwell 24 culture dishes) were washed in PBS and re-suspended in 1 ml of Hank’s balanced salt solution (HBSS) containing 5 μM Fura 2-AM for 45 minutes. Thereafter, cells were washed with the same buffer to remove excess of Fura 2-AM and incubated in Ca^2+^-free HBSS/0.5 mM EGTA buffer for 15 minutes to allow hydrolysis of Fura 2-AM into its active-dye form, Fura 2. MIA PaCa-2 and PANC-1 cells were then transferred to the spectrofluorimeter (Perkin-Elmer LS-55). Treatment with ionomycin (1 μM), fMLP (50 nM, Sigma Aldrich), with Ac 2-26 (1 μM, Tocris Bioscience) or Boc-1 (10 μM, Bachem AG) was carried out by adding the appropriate concentrations of each substance into the cuvette in Ca^2+^-free HBSS/0.5 mM EGTA buffer. The excitation wavelength was alternated between 340 and 380 nm, and emission fluorescence was recorded at 515 nm. The ratio of fluorescence intensity of 340/380 nm (F340/F380) was used to estimate intracellular free calcium. Results are indicated as delta increase of fluorescence ratio (F340/F380 nm) induced by ionomycin - basal fluorescence ratio (F340/F380 nm).

### *In vitro*wound-healing assay

MIA PaCa-2 and PANC-1 cells were seeded in a 12-well plastic plate at 5 × 10^5^ cells per well. After 24 hours incubation, cells reached 100% confluency and a wound was produced at the centre of the monolayer by gently scraping the cells with a sterile plastic p10 pipette tip. After removing incubation medium and washing with PBS, cell cultures were incubated in the presence of fMLP (50 nM), Ac2-26 (1 μM), Boc-1 (10 μM) or in growth medium as control. In case of transfection with siANXA1s, cells were plated at a number of 2 × 10^5^, after 24 hours were transfected with siANXA1s or with scrambled siRNAs and, 72 hours after transfection, wound was produced. The wounded cell cultures were then incubated at 37°C in a humidified and equilibrated (5% v/v CO_2_) incubation chamber of an Integrated Live Cell Workstation Leica AF-6000 LX. A 10× phase contrast objective was used to record cell movements with a frequency of acquisition of 10 minutes. The migration rate of individual cells was determined by measuring the distances covered from the initial time to the selected time-points (bar of distance tool, Leica ASF software). For each condition five independent experiments were performed. For each wound five different positions were registered, and for each position ten different cells were randomly selected to measure the migration distances. Statistical analysis was performed by using GraphPad Prism software (GraphPad Software Inc., version 5.0). Data are presented as means ± SEM. Values p < 0.05 were considered as significant.

### Matrigel invasion assay

MIA PaCa-2 and PANC-1 invasiveness was studied using the Trans-well Cell Culture (12 mm diameter, 8.0-fim pore size) purchased form Corning Incorporated (USA). The chambers were coated with Matrigel (Becton Dickinson Labware) that was diluted with 3 volumes of DMEM serum-free and stored at 37°C until its gelation. Cells were plated in 350 μl of DMEM serum-free at a number of 9 × 10^4^/insert in the upper chamber of the trans-well. 1,4 ml of DMEM with or without FBS were put in the lower chamber and the trans-well was left for 24 hours at 37°C in 5% CO_2_ -95% air humidified atmosphere. After that, the medium was aspirated, the filters were washed twice with PBS 1× and fixed with 4% p-formaldehyde for 10 minutes, then with 100% methanol for 20 minutes. The filters so fixed, were stained with 0,5% crystal violet prepared from stock crystal violet (powder, Merck Chemicals) by distilled water and 20% methanol for 15 minutes. After that, the filters were washed again in PBS 1× and cleaned with a cotton bud. The number of cells that had migrated to the lower surface was counted in twelve random fields using EVOS light microscope (10×) (Life technologies Corporation).

### Statistical analysis

All results are the mean ± SEM of at least 3 experiments performed in triplicate. The optical density of the protein bands detected by Western blotting was normalized against tubulin levels. Statistical comparisons between groups were made using two-way ANOVA or unpaired, two-tailed *t*-test comparing two variables. Differences were considered significant if p < 0.05 and p < 0.01.

## Results

### Expression and localization of ANXA1 in PC cell lines

**Figure 1:**
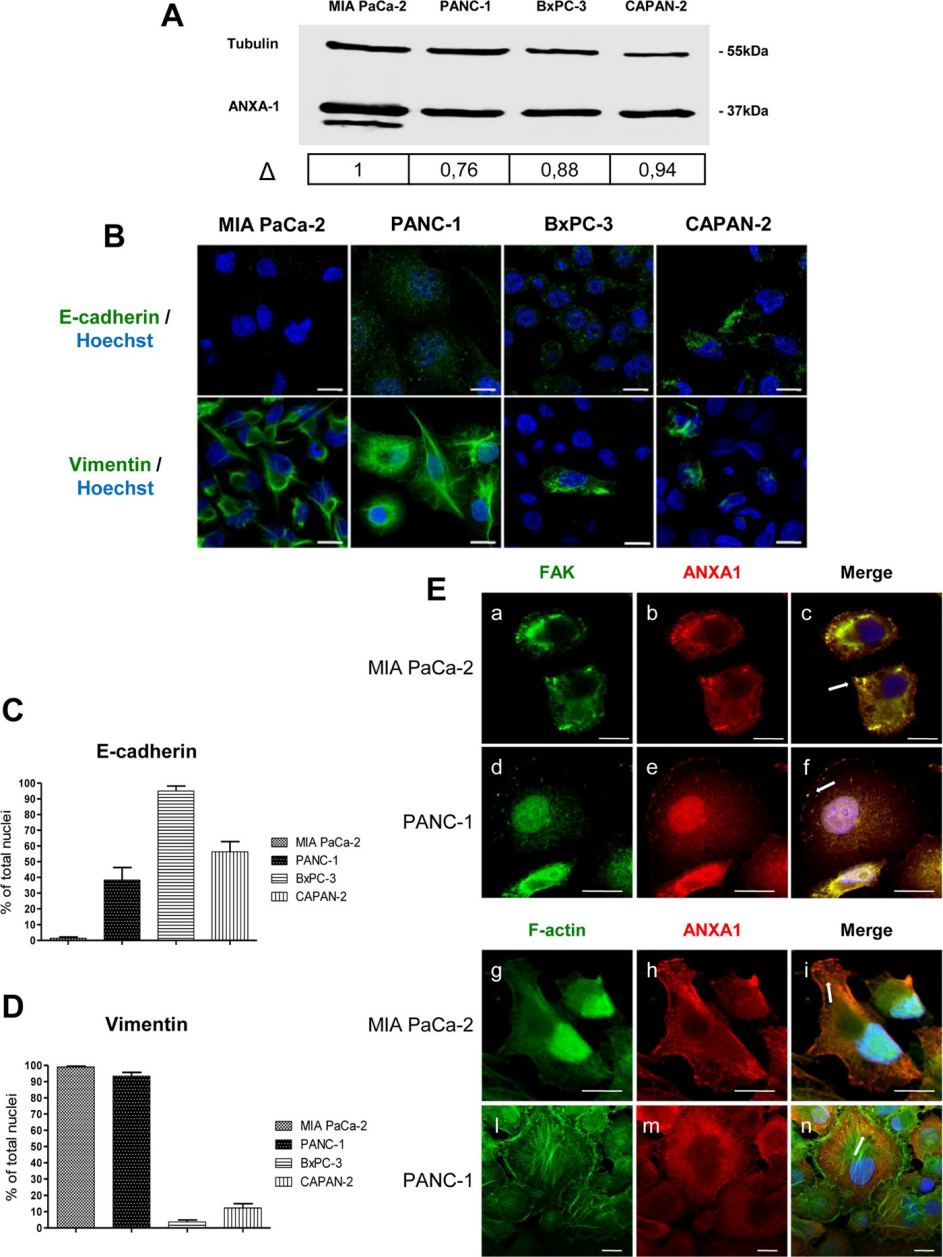
**Expression and localization of ANXA1 in human PC cells. A**, Total ANXA1 expression in MIA PaCa-2, PANC-1, BxPC-3 and CAPAN-2 cells was analyzed by Western blot with anti-ANXA1 antibody. Δ represents ANXA1 fold change by densitometry. **B**, Cultured human MIA PaCa-2, PANC-1, BxPC-3 and CAPAN-2 cells fixed and labelled with fluorescent antibody against E-cadherin and vimentin (green). **C**, **D**, Quantitative analysis of E-cadherin and vimentin protein expression in MIA PaCa-2, PANC-1, BxPC-3 and CAPAN-2 cells. Histograms represent the percentage of E-cadherin and vimentin positive cells on total DAPI positive nuclei. Results are mean ± standard deviation from three independent experiments, where at least 100 nuclei per cell line were counted. **E**, Immunofluorescence analysis to detect FAK (panels a, d), ANXA1 (panels b, e, h, m), and F-actin (panels g, l) in MIA PaCa-2 and PANC-1. Nuclei were stained with DAPI. The merged image shows overlapping localization of the proteins (panels c, f, I, n). Magnification 63×. The data are representative of 5 experiments with similar results. Bar = 10 μm.

ANXA1 role in PC progression is poorly described. Therefore, we initially focused to define how ANXA1 is expressed and localized in several human PC cell lines as MIA PaCa-2, PANC-1, BxPC-3 and CAPAN-2. These cells show many differences about their genotype, such as Kras or p16 mutations, and phenotype, as adhesion, migration, invasion and angiogenesis capacities [26]. As shown in Figure 1A, Western blotting analyses revealed that all cell lines expressed ANXA1.

Next, we characterized MIA PaCa-2, PANC-1, BxPC3 and CAPAN-2 cells on the basis of their phenotype since the more aggressive and invasive cancer cells had a higher basal Epithelial-Mesenchymal Transition (EMT) signature [27]. Confocal microscopy analyses confirmed, as previously described [26], more aggressive features for MIA PaCa-2 and PANC-1 as, differently from BxPC-3 and CAPAN-2, these cancer cells possess a marked mesenchymal phenotype characterized by up-regulation of the mesenchymal marker vimentin and down-regulation of the epithelial marker E-cadherin (Figure 1B,C,D) [28].

Tumour cell invasion and metastasis processes involve many proteins that are required for normal cell motility. As it is known that ANXA1 plays a role in normal cell migration [12, 13] and in cancer cell invasion and metastasis [14, 10], we also analyzed by confocal microscopy ANXA1 localization in the cellular motility structures identified by using focal adhesion kinase (FAK) or F-actin staining. In the figure 1E (panels c, f) we show that ANXA1 co-localized in both MIA PaCa-2 and PANC-1 cells with FAK, a protein commonly expressed in adhesion hot spots of migrating/invasive cells. Moreover, we show an actin-like filamentous ANXA1 organization and an enrichment of the protein at burble ends and extrusions in MIA PaCa-2 cells (Figure 1E, panel i). Also in PANC-1 cells ANXA1 co-localized with F-actin protein although this cell line is characterized by a less mesenchymal-like phenotype (Figure 1E, panel n).

### Effects of ANXA1 knockdown on MIA PaCa-2 and PANC-1 cell migration and invasiveness

As it is known from previous reports and confirmed by our data using confocal microscopy, PANC-1 and MIA PaCa-2, differentially from CAPAN-2 and BxPC-3, show a more aggressive phenotype, particularly MIA PaCa-2 that have an higher tumorigenic potential [29, 26].

**Figure 2:**
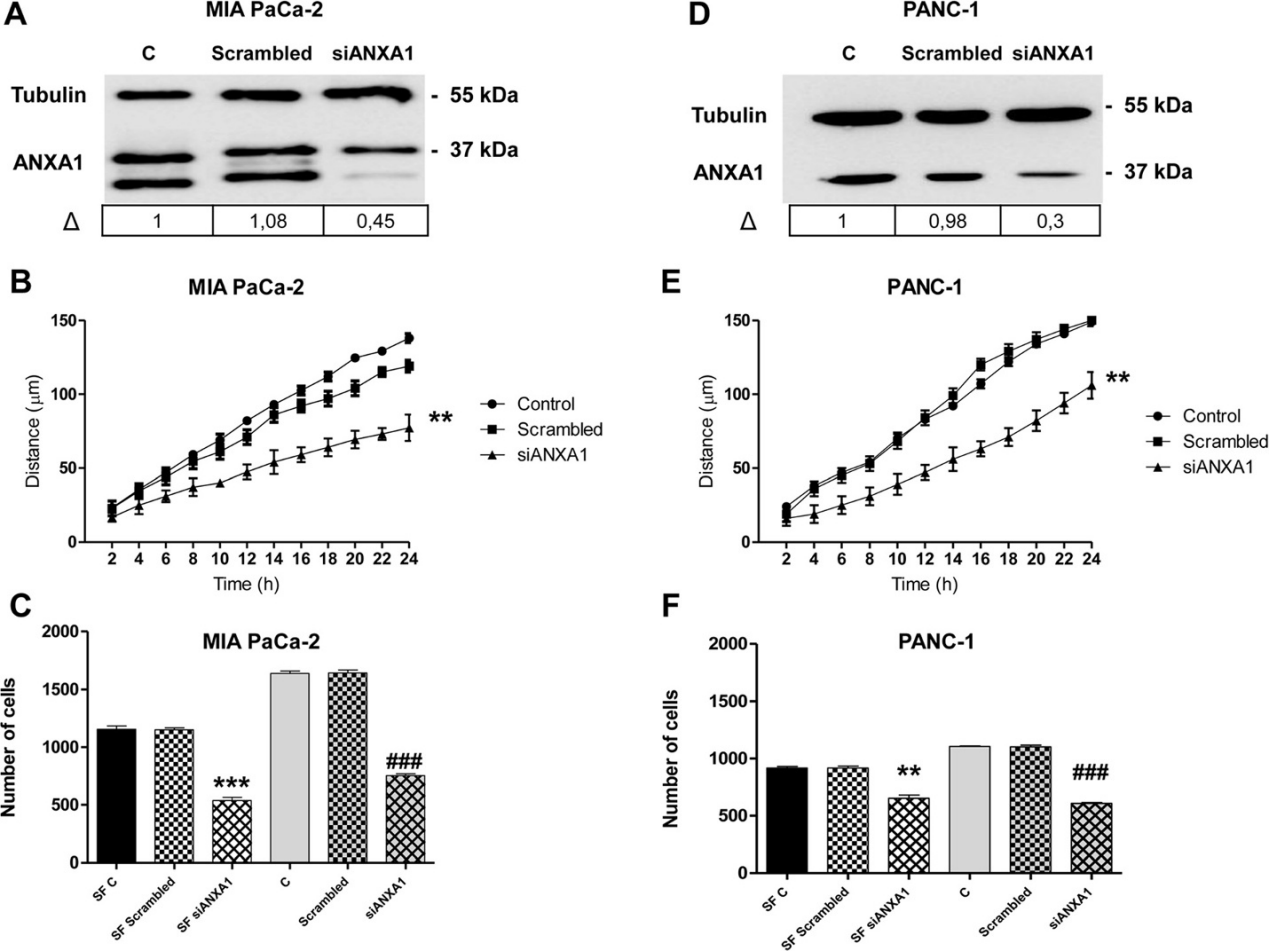
**Effects of ANXA1 knockdown on migration and invasiveness rate of MIA PaCa-2 and PANC-1 cells.** Western blot using an anti-ANXA1 antibody on protein extracts from MIA PaCa-2 (panel **A**) or PANC-1 cells (panel **D**) treated or not with siRNAs direct against ANXA1 (siANXA1). Δ represents ANXA1 fold change normalized to control levels by densitometry. Protein normalization was performed on tubulin levels. **B**, Results of wound-healing assay on MIA PaCa-2 (panel **B**) or PANC-1 cells (panel **E**) transfected with siANXA1s or scrambled siRNAs. Statistical significance was calculated using unpaired *t*-test between control and ANXA1 knock-down cells, **p < 0.01 vs untreated control. The data are representative of 5 independent experiments ± SEM. Invasiveness rate of MIA PaCa-2 (panel **C**) or PANC-1 cells (panel **F**). In invasion assays a total of 90,000 cells were transfected or not with siANXA1s (5nM) or scrambled siRNAs (5nM) for 72 h and plated as described in Methods section. Invasiveness rate was founded out by counting stained cells on the lower surface of the filters. Data represent mean cell counts of 12 separate fields per well ± SEM of 5 experiments. Statistical significance was calculated using unpaired *t*-test between control and ANXA1 knock-down cells **p < 0.01 vs serum free (SF) control; ***p < 0.001 vs SF control; ^###^p < 0.001 vs control.

We observed that in MIA PaCa-2 and PANC-1 cell lines ANXA1 localized in the regions that are involved in the cell movement. As the migration/invasion processes start once cells form actin- and FAK-rich protrusions that adhere to the matrix and create the tension forces necessary for cell motility [30], we hypothesised a role for the protein in these processes. The expression of ANXA1 was greatly reduced in MIA PaCa-2 (Figure 2A) and PANC-1 (Figure 2D) cells by specific siRNA transfection. Thus a wound-healing migration assay on cellular monolayer in ANXA1 knockdown cells was performed. The confluent cultures were scraped to create a wound and cell migration was monitored by time-lapse video-microscopy at the site of the wound. We measured the migration distances of selected cells at different time points as described in Methods section. In ANXA1 knockdown MIA PaCa-2 (Figure 2B) and PANC-1 (Figure 2E) cells the rate of migration decreased in a significant manner, if compared with the wild type control and with scrambled RNA transfected cells.

The matrigel invasion assay was also performed in ANXA1 knockdown MIA PaCa-2 and PANC-1 cells to investigate the role of ANXA1 on their invasion ability. As shown in Figure 2C and 2F, siRNAs against ANXA1 markedly suppressed the invasiveness of both PC cell lines. To confirm the technical efficiency of our experiment, we used a serum free control to eliminate any chemoattractant condition: in this way we found significantly less invading cells on the lower surface of matrigel (data not shown).

### Localization and cleavage of endogenous ANXA1 in MIA PaCA-2 cells

Cellular migration and invasion events can be triggered by a number of molecular signals, such as chemoattractants and mechanical forces, which are sensed by receptors on the cell surface or within cells to lead to a migratory response [31].

The extracellular form of ANXA1 has been described to play a role in cancer cell invasion and metastasis. Although the protein does not possess classical signal sequences to target the protein for export, both the full-length and truncated forms are often observed in extracellular environments. Moreover, it appears that proteolytic cleavage of ANXA1 is required for protein secretion, because the majority of ANXA1 released from neutrophils is N-terminally cleaved [32–38]. Based on this information, our characterization experiments continued with the analysis by Western blot of ANXA1 expression in sub-cellular compartments of MIA PaCa-2 cells. In particular, we obtained membrane, cytosol and nuclear protein extracts as described in Methods section. The ANXA1 membrane expression was detected by both fractioned protein extracts and EDTA wash, with which we obtained the proteins that bind plasma membrane through calcium. In MIA PaCa-2 extracts, we found both, full length (37 kDa) and cleaved (33 kDa) forms of ANXA1 protein at plasma membrane and in the cytosol but not in the nucleus where only the 37 kDa ANXA1 form was expressed. Conversely, PANC-1 didn’t show the ANXA1 cleaved form and the protein expression in sub-cellular compartments was characterized by a small amount onto membrane and in the nucleus, if compared with MIA PaCa-2 cells.

**Figure 3:**
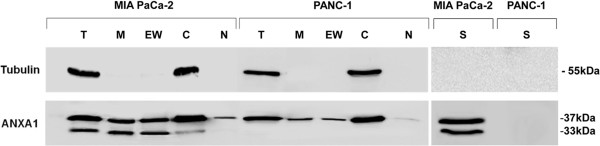
**Expression and localization of cleaved and secreted form of ANXA1 from MIA PaCa-2 and PANC-1 cells.** Cellular compartments were obtained as described in Methods section. Total (T), membrane (M), EDTA Wash (EW), cytosolic (C), nuclear (N) and supernatant (S) ANXA1 expression in protein extracts from MIA PaCa-2 and PANC-1 was examined by Western blot with anti-ANXA1 antibody. The protein bands were normalized on tubulin levels. The data are representative of 5 experiments with similar results.

Both full length (37 kDa) and cleaved (33 kDa) forms of ANXA1 were also observed in MIA PaCa-2 supernatants, whereas no protein secretion was observed in the PANC-1 supernatants (Figure 3).

### Expression and activation of FPRs in MIA PaCa-2 and PANC-1 cells

Cancerous cell capability to migrate and invade tissues is a decisive aspect of cancer progression and entails the coordination of several cellular events, such as cytoskeletal reorganization, dynamic cell-matrix adhesion and remodelling [30].

Regulatory action on cell surface by extracellular ANXA1 is reported to be mediated by signalling through FPRs [12-13; 20,21] that are supposed to regulate cell migration by actin polymerization.

**Figure 4:**
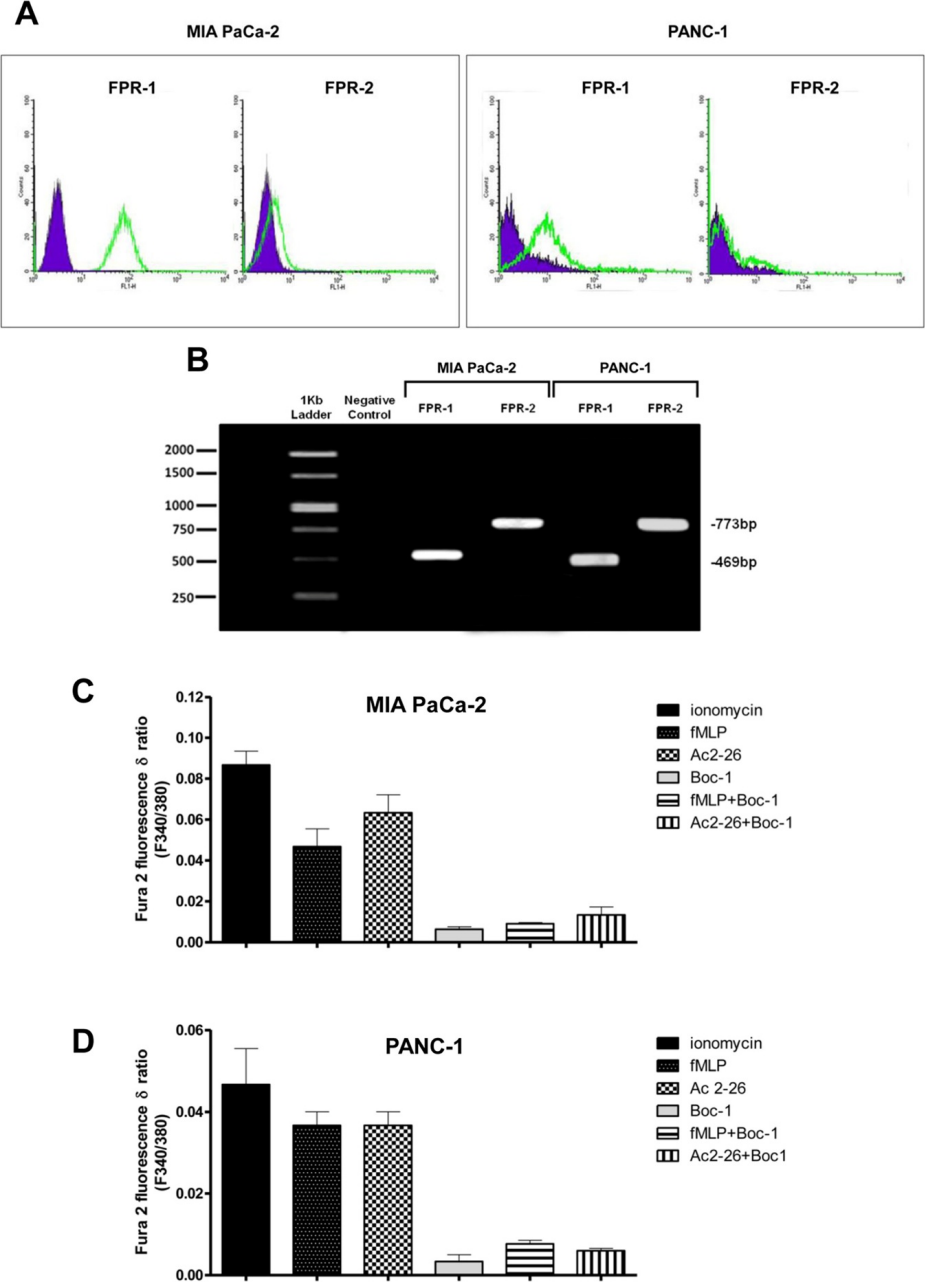
**FPR-1 and 2 expression and activation in MIA PaCa-2 and PANC-1 cells. A**, Cell surface expression of FPR-1 and FPR-2 in MIA PaCa-2 and PANC-1 cells was analyzed by flow cytometry. The violet areas in the plots are relative to secondary antibody alone. FPR-1 and FPR-2 signals are showed with green bends. **B**, Expression of FPR-1 and FPR-2 in MIA PaCa-2 and PANC-1 cells analyzed by PCR. Effects of fMLP (50 nM), Ac-2-26 (1 μM) and FPR pan-antagonist Boc-1 (10 μM) on the FPR-induced rise in intracellular Ca^2+^ in MIA PaCa-2 (panel **C**) or PANC-1 cells (panel **D**). Cells were treated as described in Methods section. The histograms show the fluorescence ratio calculated as F340/F380 nm in absence of extracellular Ca^2+^. Control represents ionomycin-stimulated cells. Data are means ± SEM (n = 5).

In order to verify the role of ANXA1-FPR interaction in MIA PaCa-2 and PANC-1 cell migration and invasiveness, we first assessed FPR expression in these cell lines by cytofluorimetric analysis (Figure 4A): we found that FPR-1 and FPR-2 were expressed in both cell lines. These findings were confirmed by qualitative PCR (Figure 4B).

It is known that the interaction between ANXA1 and FPRs causes a series of cellular responses, such as the ERK phosphorylation and the increase of intracellular [Ca^2+^] concentration. The N-terminal mimetic peptide of ANXA1, Ac2-26, can activate all three human FPRs, promoting calcium fluxes, and cell locomotion. To determine whether ligand binding to FPRs induces similar signal transduction in MIA PaCa-2 and PANC-1, we examined the stimulated release of calcium from intracellular stores. Cells were incubated in Ca^2+^ free medium and loaded with the fluorescent calcium indicator FURA-2 AM before stimulation with Ac2-26 (1 μM) or the natural FPR agonist fMLP (50 nM) together or not with the FPR pharmacological antagonist Boc-1 (10 μM) that is able to antagonize all three human FPR isoforms. The spectrofluorimetric assay (Figure 4, panels C and D) shows that fMLP and peptide Ac2-26 were able to increase the mobilization of intracellular Ca^2+^ in both MIA PaCa-2 and PANC-1 cells. In fact, no significant differences between ionomycin (used as reference compound) and fMLP or Ac2-26 were observed. The effects of fMLP and Ac2-26 peptides were inhibited by the pharmacological pan-antagonist Boc-1 in both cell lines.

### Effects of extracellular ANXA1 on MIA PaCa-2 and PANC-1 cells

To determine if ANXA1 influences cell migration acting through FPRs, we performed a wound-healing migration assay on cellular monolayer in both the analyzed cell lines.

**Figure 5:**
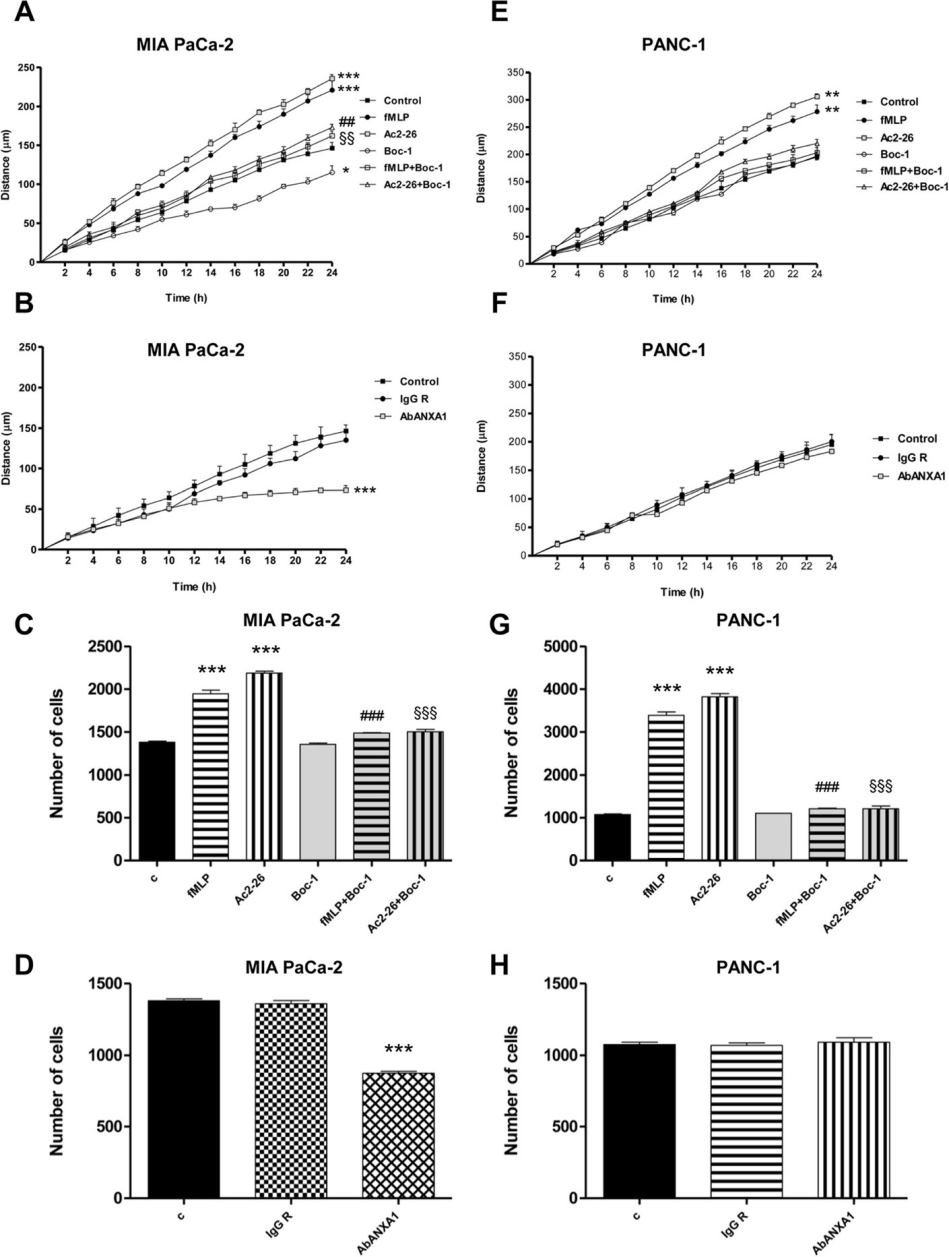
**Wound-healing and matrigel invasion assay on MIA PaCa-2 and PANC-1 cells.** Wound-healing assay on MIA PaCa-2 (panel **A**) or PANC-1 cells (panel **E**) treated or not with fMLP (50nM), Ac2-26 (1 μM), Boc-1 (10 μM), fMLP + Boc-1 or Ac2-26 + Boc-1; Statistical significance was calculated using two way ANOVA,* p < 0.05, **p < 0.01, ***p < 0.001 vs untreated control; ^##^p < 0.01 and ^§§^p < 0.01 vs respective controls. Wound-healing assay on MIA PaCa-2 (panel **B**) or PANC-1 cells (panel **F**) treated or not with ANXA1 blocking antibody (AbANXA1) or scrambled rabbit IgG (IgG R); Statistical significance was calculated using unpaired *t*-test between control and treated cells, ***p < 0.001 vs untreated control. All wound-healing data are representative of 5 experiments ± SEM. Invasiveness rate of MIA PaCa-2 (panel **C**) or PANC-1 cells (panel **G**) treated or not with fMLP (50 nM), Ac2-26 (1 μM), Boc-1 (10 μM), fMLP + Boc-1 or Ac2-26 + Boc-1; Statistical significance was calculated using two way ANOVA, ***p < 0.001 vs untreated control; ^###^p < 0.001 and ^§§§^p < 0.001 vs respective controls. Invasiveness rate of MIA PaCa-2 (panel **D**) or PANC-1 cells (panel **H**) treated or not with AbANXA1 or IgG R; Statistical significance was calculated using unpaired *t*-test between control and treated cells, ***p < 0.001 vs untreated control. Invasiveness rates were measured as described in Methods section. Data represent mean cell counts of 12 separate fields per well ± SEM of 5 independent experiments.

For MIA PaCa-2, results in Figure 5A showed an increase in migration speed of cells treated with Ac2-26 (1 μM) or fMLP (50 nM) compared to control cells. The FPR pan-antagonist Boc-1 (10 μM) significantly inhibited basal and stimulated migration. Interestingly, an ANXA1 blocking antibody was able to reduce in a significant manner MIA PaCa-2 cell migration (Figure 5B).

The role of ANXA1 in cancer progression is still discussed; this protein may have specific functions in different tumoral models. For example, in gastric and colon carcinomas ANXA1 has a pro-invasive role through its interaction with FPRs [15, 21].

When treated with Ac2-26 (1 μM) and fMLP (50 nM), MIA PaCa-2 cells showed an increased invasion speed through coating of matrigel. Again, Boc-1 antagonist (10 μM, Figure 5C) reduced in a significant manner MIA PaCa-2 stimulated cell invasion. An ANXA1 blocking antibody was also able to inhibit basal cell invasion (Figure 5D).

At the same time, we used PANC-1 cell line as basis for comparison, as these cells did not show either the ANXA1 cleaved form or the externalized one. Similarly for MIA PaCa-2, results of the wound-healing migration assay on cellular monolayer showed an increase in migration speed of the cells treated with Ac2-26 (1 μM) or fMLP (50 nM) when compared to control cells and a reverted effect in cells treated with the pan-antagonist Boc-1 (10 μM) (Figure 5E). Interestingly, ANXA1 blocking antibody had no effects on PANC-1 cell motility (Figure 5F). In order to verify whether the absence of extracellular ANXA1 in PANC-1 cell line was also implied in a different invasiveness rate, we performed a matrigel invasion assay. When treated with Ac2-26 (1 μM) and fMLP (50 nM), PANC-1 cells showed a large invasion speed through coating of matrigel. In both of cases, experimental points were compared with non treated control or with Boc-1 antagonist (10 μM) treated cells (Figure 5G). ANXA1 blocking antibody had no effects on PANC-1 invasiveness (Figure 5H).

### Effects of MIA PaCa-2 supernatants on PANC-1 cell migration

**Figure 6:**
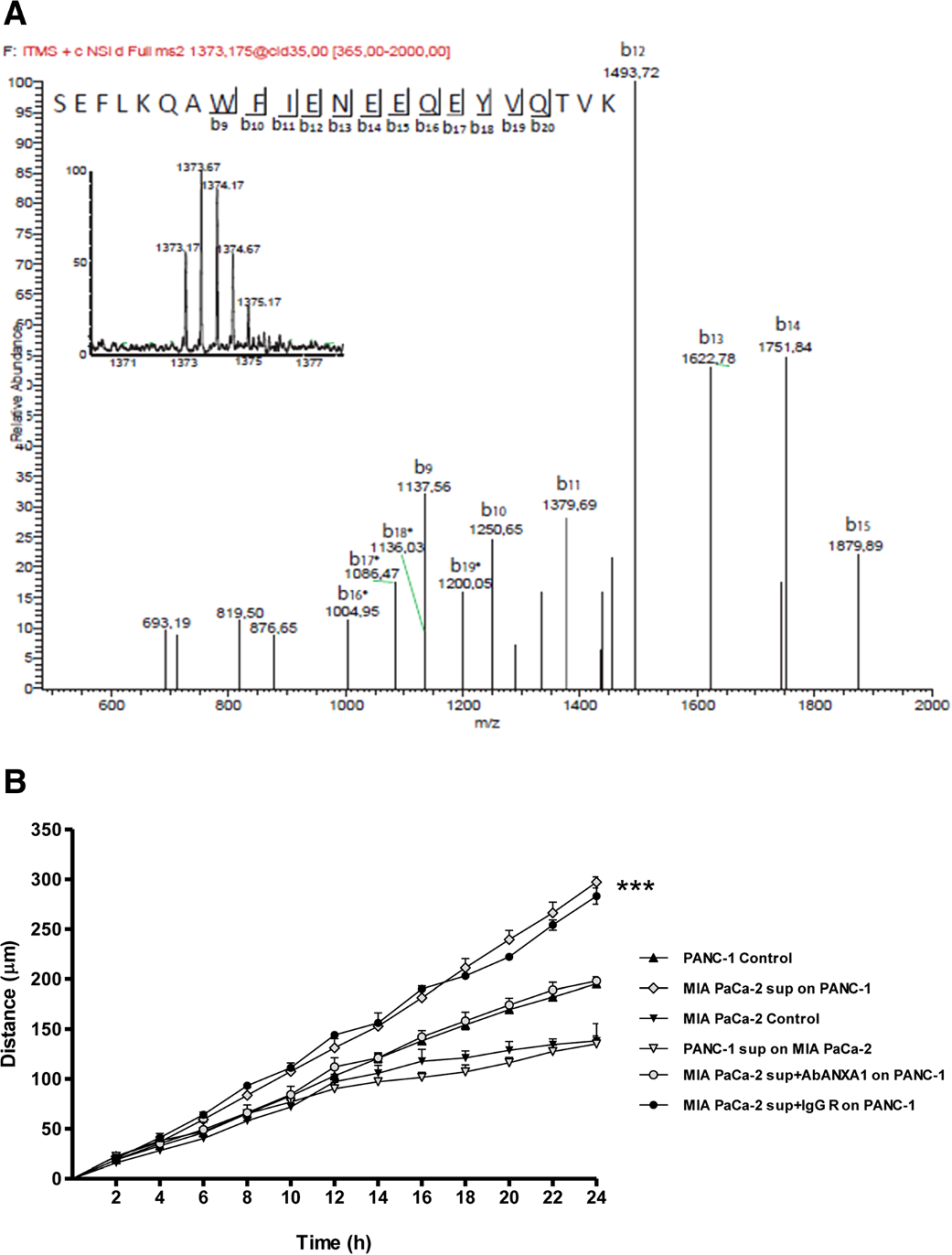
**Analysis of the secreted forms of ANXA1 and of their effects on PC cell motility. A**, LC-HRMS/MS spectrum, peaks refer to all the discovered peptide fragments. The data are representative of 5 experiments with similar results. **B**, Effects of MIA PaCa-2 supernatants on PANC-1 cells and *viceversa* in a wound-healing assay. Statistical significance was calculated using unpaired *t*-test between control and treated cells, ***p < 0.001. Data are means ± SEM (n = 5).

Our data suggest a double role of ANXA1 in PC cell motility. In one way the protein acts in the intracellular environment thanks to its involvement in cytoskeleton reorganization, as confirmed by immunofluorescence assay showed in Figure 1E. In another one, ANXA1 externalized form appears to bind FPRs and trigger some molecular pathways that lead to cell migration and invasion. Based on these findings, we focused on the appearance of a 33 kDa form only in MIA PaCa-2 cells, both in total (Figure 1A) and supernatant extracts (Figure 3). Therefore, in order to detect possible ANXA1 fragments released from the cells a multi-step fractionation of MIA PaCa-2 supernatants was performed. Obtained samples were analyzed by LC-HRMS/MS as described in Methods section. A peptide showing a molecular weight of 2744.324 was detected; on the basis of its molecular weight and of the CID induced fragmentation spectrum (Figure 6A), this peptide was identified as the fragment 4-26 of ANXA1.

Furthermore, we considered the higher migratory and invasive rate of MIA PaCa-2 compared with PANC-1 cells [26]. In order to confirm that the secreted forms of ANXA1 protein were able to induce PC cell migration and invasion in autocrine and paracrine manner, we performed further experiments adding MIA PaCa-2 supernatants to PANC-1 cells and *viceversa*. As shown in Figure 6B, MIA PaCa-2 supernatants containing all the secreted forms of ANXA1 protein (37 kDa, 33 kDa and 3 kDa) significantly increased PANC-1 cell migration rate. Conversely, the administration of PANC-1 supernatants on MIA PaCa-2 cells had no effects on migration speed of the latter ones. Moreover, the administration of MIA PaCa-2 conditioned supernatant containing ANXA1 blocking antibody on PANC-1 cells did not increase the migration rate of these cells.

## Discussion

The role of ANXA1 in tumours is paradoxical since ANXA1 appears to behave either as a tumour suppressor or an oncogenic gene. As the mechanism of ANXA1 in cancer progression has not been still completely clarified, more studies are required to investigate the detailed action mechanisms of this protein in tumours. Accumulated evidences have indicated that ANXA1 deregulation and sub-cellular localization are involved in the development, invasion, metastasis and drug resistance of a variety of cancers suggesting a tissue type-specific role for ANXA1 in tumour advancing [9]. In particular, concerning cellular motility, ANXA1 actions are exerted extracellularly via FPRs in autocrine/paracrine manner, but also in the intracellular environment where it contributes to the dynamic reorganization of the actin cytoskeleton [11].

It has been shown that ANXA1 over-expression in the tissues from patients with PC is correlated with poor differentiation and prognosis and seems to be associated with malignant transformation and cancer progression [39–42].

In the present paper, we report that ANXA1 could have a role in PC cell migration and invasiveness and should be involved in the metastatic capability of these cells.

We first analyzed ANXA1 expression in MIA PaCa-2, PANC-1, BxPC-3 and CAPAN-2 PC cell lines and we found that all of them expressed high levels of ANXA1.

Moreover, all analyzed PC cell lines showed at least two different phenotypes: a less aggressive epithelial-like and a more aggressive mesenchymal-like. In the latter, ANXA1 was mainly localized in the regions involved in cellular motility, suggesting an intracellular role for the protein in the processes of cell migration/invasion.

Given the less aggressive phenotype of CAPAN-2 and BxPC-3 cells, we chose to use only MIA PaCa-2 and PANC-1 cells that present more marked mesenchymal features. In particular, among the other PC cell lines, MIA PaCa-2 cells are commonly used to induce tumour xenografts in nude mice because of their strong capability to develop not only the largest tumoral mass but also metastasis [26].

Actually, the inhibition by siRNAs of ANXA1 expression in both MIA PaCa-2 and PANC-1 cells induced a significant decrease of the migration rate and markedly suppressed the invasiveness of these cells, confirming that intracellular ANXA1 is involved in PC cell migration/invasion.

ANXA1 has been shown to differently localize to the cellular compartments of various cell types including leukocytes, endothelial cells, lung epithelial cells and synoviocytes where it is thought to be important in biological functions [43–47]. Moreover, recent studies have shown that, in PC tissues, localization of ANXA1 was different in cytoplasm and membrane of tumour cells, indicating that the function of ANXA1 may vary among its differential localization.

We found that both MIA PaCa-2 and PANC-1 cells exhibit membrane and nuclear ANXA1 expression. ANXA1 membrane translocation has been reported to be strictly related to both membrane trafficking and secretion of the protein in extracellular environments and to be leaded by post-translational modifications [32]. Concerning the exact physiological functions of nuclear-translocated ANXA1 protein, accumulated evidence like in oral squamous cell carcinoma, gastric carcinoma and oesophageal squamous cell carcinoma, indicate that ANXA1 nuclear expression correlates with advanced disease and cancer dissemination. However, the mechanisms underlying ANXA1 nuclear translocation remain unknown [48–50].

We also found that MIA PaCa-2 cells exhibit increased extracellular ANXA1, so that we have hypothesized a role for the secreted protein in regulating PC cell migration/invasion through the interaction with FPRs.

FPRs are expressed in several cellular populations and bind a variety of exogenous and endogenous ligands that elicit differential biological responses [16]. ANXA1 and its N-terminal mimetic peptide, Ac2-26, are endogenous FPR ligands. Flow cytometry and PCR analyses showed that MIA PaCa-2 and PANC-1 cells express FPR-1 and FPR-2. Moreover, experiments on the mobilization of intracellular calcium have confirmed the activity of the FPRs in these cell lines, following stimulation with the Ac2-26 peptide. We found no receptor activation in presence of Boc-1, a molecule that at a dose of 10 μM can be considered as able to block all the three receptor isoforms [16].

Exogenous administration of Ac2-26 was also able to increase migration speed and invasiveness of cells through coating of matrigel, compared to relative controls. The specificity of Ac2-26-induced effects on wound closure and invasiveness through the FPRs was confirmed by administration of the FPR pan-antagonist Boc-1. The same results were obtained with ANXA1 blocking antibody, able to reduce PC cell motility in MIA PaCa-2 cells. Our results are consistent with the observed role of ANXA1 in head neck squamous cancers where the protein over-expression was associated with increased tumour invasiveness and metastasis and in SK-CO15 intestinal epithelial cells where ANXA1 regulated cellular invasive behaviour acting through FPRs [51, 21].

Differently from MIA PaCa-2, no secreted forms of ANXA1 protein were observed in protein supernatant extracts from PANC-1 cells, consequently, we reasoned on the role of the extracellular protein in PC cell line migration and invasiveness. Results of a wound-healing migration assay on PANC-1 cells showed an increase in migration and invasiveness rate of cells treated with Ac2-26: these findings are consistent with the expression of FPR-1 and FPR-2 that we found in these cells. However, we showed that ANXA1 blocking antibody had no effects on PANC-1 cell motility.

We also report the presence of the full-length form (37 kDa) accompanied by the appearance of the 33 kDa cleavage product of ANXA1 in several cellular compartments of MIA PaCa-2 cells. Furthermore, these two forms are secreted outside the cells, since they appeared in cellular supernatants. LC-HRMS/MS, used to characterize secreted forms of ANXA1, showed a peptide with molecular weight of 2744.324 demonstrating, for the first time, the presence of the fragment 4-26 of ANXA1 in the extracellular environments.

In PC there is an abnormally high expression of a number of important tyrosine kinase growth factors and receptors, like the Epidermal Growth Factor (EGF) family, which may contribute to the neoplasia growth by autocrine and paracrine effects [40, 41]. Immunohistochemistry studies showed that EGF Receptor (EGFR) over-expression positively correlates with advanced tumour staging and lymph node metastasis [42]. ANXA1 is a substrate protein of EGFR, so it can be postulated that constantly activated EGFR pathway could promote ANXA1 up-regulation and post-transductional modifications, which might be associated with its membrane translocation and secretion [52].

ANXA1 cleavage and secretion could be also mediated by several proteinases [53]: differential expression or activity of these enzymes could explain the lack of both ANXA1 cleavage and secretion in PANC-1 cells. However, further studies will be needed to address this point.

As previously reported, MIA PaCa-2 have a more invasive behaviour than PANC-1 cells [54, 55] and we suppose that these differences could be due to the presence of secreted forms of ANXA1 protein. The addition of MIA PaCa-2 supernatant to PANC-1 cells significantly stimulated PANC-1 migration rate. Conversely, PANC-1 supernatant administration on MIA PaCa-2 cells had no effects on cell motility, confirming that the secreted forms of ANXA1 protein may be able to induce PC cell migration and invasion.

The therapeutic properties of ANXA1 on wound closure are well known in other tissues and could be due to the autocrine and paracrine anti-inflammatory effects of its N-terminus peptide, Ac2-26, that is released from the full length protein through regulated proteolysis [56]. It has recently shown that both the full length protein and the Ac2-26 peptide promote cell motility in intestinal epithelial cells through FPRs [57]. Our studies are consistent with these findings and could explain the biological effects of ANXA1 and its cleavage products on the ability of MIA PaCa-2 cells to have a more invasive behaviour.

## Conclusions

In summary, our data suggest a role for intracellular and extracellular ANXA1 in stimulating PC cell migration and invasion. However, future studies on PC patients will be needed to determine whether ANXA1 could represent a potential diagnostic, prognostic or predictive biomarker by correlating the progression and/or metastatic rate of the tumour to the protein expression.
